# Motion velocity as a preattentive feature in cartographic symbolization

**DOI:** 10.16910/jemr.16.4.1

**Published:** 2023-09-14

**Authors:** Paweł Cybulski, Vassilios Krassanakis

**Affiliations:** Adam Mickiewicz University, Poznań, Poland; University of West Attica, Egaleo (Athens), Greece

**Keywords:** Preattentive processing, gaze, map perception, animated mapping, eye movement, dynamic cartographic symbols, eye tracking, attention

## Abstract

The presented study aims to examine the process of preattentive processing of dynamic point symbols used in cartographic symbology. More specifically, we explore different motion types of geometric symbols on a map together with various motion velocity distribution scales. The main hypothesis is that, in specific cases, motion velocity of dynamic point symbols is the feature that could be perceived preattentively on a map. In a controlled laboratory experiment, with 103 participants and eye tracking methods, we used administrative border maps with animated symbols. Participants’ task was to find and precisely identify the fastest changing symbol. It turned out that not every type of motion could be perceived preattentively even though the motion distribution scale did not change. The same applied to symbols’ shape. Eye movement analysis revealed that successful detection was closely related to the fixation on the target after initial preattentive vision. This confirms a significant role of the motion velocity distribution and the usage of symbols’ shape in cartographic design of animated maps.

## Introduction

Cartographic visualization is one of the most popular methods of geographic data presentation. It is considered a procedure of converting a spatial database into a map (22). Cartographic symbolization is the fundamental part of visualizing spatial data. The process is based on the utilization of the visual variables proposed by Bertin (1). These are basic graphical elements that visually differentiate one object from another on a map: size, shape, value (lightness), color hue, orientation, texture, and position. However, these variables originally referred to static maps. Nowadays, animated maps constitute a common part of news information services that are the basic form of weather data presentation, historical education, video games, and also governmental websites, such as Scientific Visualization Studio NASA (4; 14). Therefore, the development of animated techniques enriches traditional visual variables and establishes new forms of cartographic symbolization called dynamic symbols (28).

The main advantage of dynamic symbols is that they enable the presentation of quantitative spatial data with variables that can be considered qualitative. For example, one could quantify a specific phenomenon on a map with a size of a circle. However, it would be ineffective if one would attempt to quantify the same data with the orientation of a rectangle. Therefore, animated techniques, by adding continuous rotation around the geometric center of the geometric figure, enable data quantification through the rotation velocity.

The velocity of motion was recognized as a preattentive feature (15). Preattentive features are attributes automatically perceived across the visual field by the low-level visual system. There is a clear evidence that although some features are so elementary to the visual system that they do not require attention, attention can be crucial in preattentive processing (18). This early stage of visual processing is performed in the first milliseconds (11; 19) and constitutes the basic concept of the Feature Integration Theory by Treisman and Gelade (35), and cannot be decomposed into simpler features (40). However, the differences in motion velocity of individual map symbols could be insufficiently large to be perceived preattentively. According to Duncan and Humphreys (9), to decrease the visual search time of the target object and to perceive it preattentively, the difference in the target feature and distractor has to be large enough. However, the visual search system is a combination of bottom-up and top-down processing (37). Based on the Guided Search Theory (38), the bottom-up search occurs after feature categorization within the initial preattentive vision. It is focused on the difference between the target object and the distractors. However, searching for graphically and dynamically similar map symbols requires a top-down process. Therefore, the dynamic point symbol on a map can be detected in a preattentive vision if its velocity of motion is unique among distractors or if visual attention could be “guided” to find the target symbol in a serial search.

The aforementioned vision theories have a great influence on cartography. Some cartographers distinguished between several types of dynamic symbol behavior, including rotation, pulsation, and blinking (flickering) (5; 28). There was an attempt to incorporate dynamic symbols in the Geographic Information Systems (GIS) software (41). The differences in velocity of motion made it possible to distinguish faster and slower symbols. Those refer to higher or lower value of the presented quantitative data. However, the studies mentioned included only geometric symbols. Map design often uses pictorial symbols (e.g., Google Maps, Bing Maps). So far, these have not been considered dynamically. Researchers experimented with searching for the target symbol among distractors in a map environment. Lloyd (29) examined the search time of unique targets and targets that share features with other symbols. Apart from visual variables, location also played a significant part in the reaction time. Michaelidou et al. (31) examined the effect of preattentive attributes of shape of point map symbols. They found that symbol with a hole would be the most efficient in searching despite the location. However, their study did not include dynamic map symbolization.

Velocity of motion was detected by several studies as a preattentive feature (36; 33; 8; 16). Therefore, there is a need to examine this attribute in cartographic research, including dynamic symbolization. The understanding of whether dynamic symbols work as preattentive features, as well as how this could impact the target location detection, in designing effective animated maps, is of fundamental significance.

Modern cartographic research refers not only to Earth sciences but also to psychology of vision (12; 30). Interdisciplinary approach, including cartographic methodology and vision studies, could bring significant contribution to both fields. For the visual search studies it would be crucial to define parameters of the velocity of motion (symbol change speed) of geometric symbols, which would enable preattentive processing on a map. In terms of cartographic research it is crucial to study the perception of dynamic symbols for effective map design. The effectiveness lies in the users’ accurate geometric symbol detection.

The examination of different concepts related to cartographic design and geographic information communication is mainly based on empirical research studies (34) that use typical experimental techniques and methods adapted by related domains, such as psychology and neuroscience (20). Among the aforementioned techniques and methods, eye tracking and eye movement analysis seem to offer great opportunities to examine several aspects related to the map reading process. Over the last years, several review studies summarized the importance of eye tracking experimentation in cartographic research (21; 23, 24) recognizing and also highlighting potential trends and research gaps towards future research (24).

The influence of preattentive vision on map reading processes constitutes one of the concepts that have been examined by means of eye tracking techniques in cartographic research. More specifically, previous studies investigated how this effect could influence both the effectiveness and efficiency of visual and dynamic variables (3; 26; 27, 25; 7). The produced results of such studies could feed the process of cartographic design directly since they had revealed fundamental functions related to map users' perception and cognition.

The research hypothesis of the presented work is that the velocity of motion of dynamic point symbols is the feature that could be perceived preattentively on a map. However, the map symbol, related motion type, and motion velocity distribution could affect the preattentive processing. Therefore, we assume that the type of motion is a significant factor in map perception. We aim to determine the motion parameters that enable preattentive processing and guide towards accurate detection of the geometric map symbols. Detection accuracy can be confirmed by the exact coordinates of the mouse cursor’s activity.

## Methods

In order to examine the research hypothesis, we designed and conducted an eye tracking study. More specifically, the experimental study was designed for two groups. In Group 1 (G1), participants were only asked to find the fastest symbol on the entire map. In Group 2 (G2), participants were shown the target symbol every time before they watched each map’s stimuli. Our approach is similar to Lloyds’ (29) research study. However, main differences are related to the usage of symbols that are dynamic. Additionally, we have taken into account the location of the target symbols, distinguishing between central and peripheral locations. This differentiation was informed by prior research on cartographic stimuli conducted by Cybulski and Krassanakis (6).

### Participants

A hundred and three students of the Adam Mickiewicz University, Poznań, Poland, aged 19-44 participated in the experiment (the average age was 22.5 ± 4.3), 55 of them identified as men, and 48 as women. The first group (G1) consisted of 50 people aged 19-41 (on average 22.6 ± 4.4), 27 men, and 23 women. In the second group (G2) there were 53 people aged 19-44 (on average 22.3 ± 4.3), 28 men, and 25 women.

All of them had normal or corrected-to-normal vision, and none had astigmatism. Before the experiment, informed consent was obtained from all participants. Participation in the study was voluntary, participants did not receive any payment, and agreed to participate on the voluntary conditions. The institution in which the research was conducted did not require permission of the ethics commission for the study.

### Materials

We designed 42 dynamic point symbols. There were three geometric symbols (square, pentagon, triangle), and five pictorial ones (palm tree, oil rig, mine cart, factory, wind turbine). The precise count of 42 symbols is derived from the combination of three categories of geometric symbols, each representing different shapes, three velocity scales associated with these symbols, and three types of dynamic changes they depict (27 geometric symbols in total). Additionally, there are five distinct types of pictorial symbols, each paired with three velocity scales (15 pictorial symbols in total). However, as our research is still ongoing, we have decided to narrow down our analysis to focus solely on geometric symbols. Hence, in the subsequent analysis, we will be examining a total of 27 dynamic geometric symbols. Pictorial symbols require additional data processing and preparation, and will be considered in future studies.

**Figure 1.**
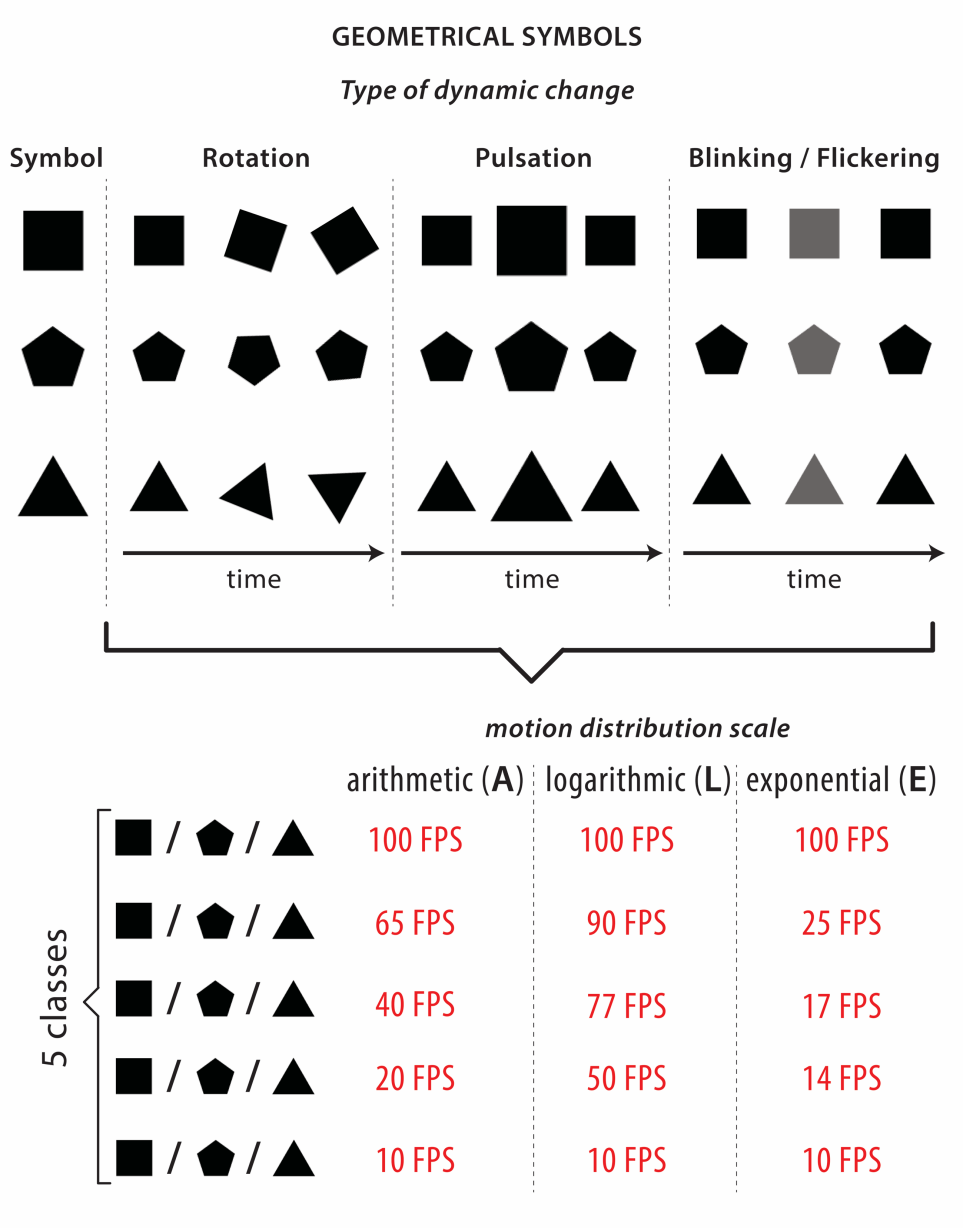
Geometric dynamic symbols were used in the experiment. Three types of dynamic changes (rotation, pulsation, blinking/flickering) are classified into five data classes according to arithmetic, logarithmic or exponential scale.

For each geometric symbol we implemented three types of dynamic changes in visual variables: rotation (continuous change of orientation around the symbols’ geometric center), pulsation (continuous change of the symbols’ size), and blinking/flickering (continuous change of brightness). We designed three scalar scales that enable motion velocity distribution. Quantitative mapping requires distribution of speed differences within symbol classes so they can be arranged from the slowest to the fastest. Following the Millers’ principle (32), and the limitations of cartographic animation perception (13), we chose five speed classes for dynamic symbols. Differences within each class were distributed in three ways: logarithmically, arithmetically, and exponentially. Figure 1 presents dynamic geometric symbol design.

In our study, we defined the motion distribution scale based on the behavior of the fastest object in the animation. For example, if the object changed its size, transparency, or orientation within 10 frames, it would complete one full cycle of change within 1 second when the animation was set at 100 fps. On the other hand, at 60 fps, the object would only complete 6 full cycles of pulsation, rotation, or blinking within the same duration.

**Figure 2.**
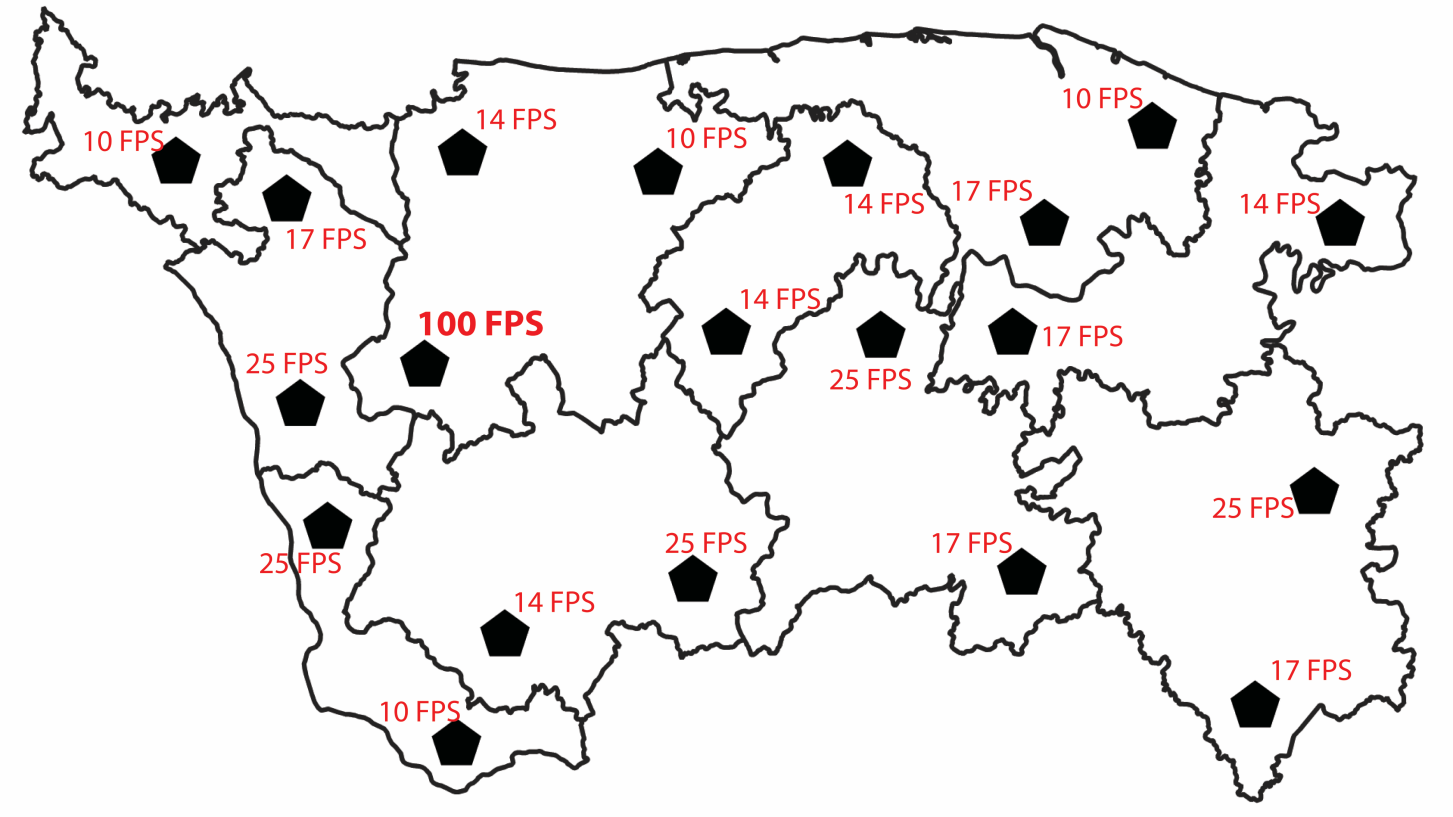
Example of administrative border map with dynamic point symbols located randomly. Red description of FPS (frames per second) was not displayed on the experimental stimuli. The map examples can be accessed here: http://kartografia.amu.edu.pl/Badanie_PC/4/Blinking_exponential_P02.html; http://kartografia.amu.edu.pl/Badanie_PC/5/Pulsation_logarithmic_T01.html; http://kartografia.amu.edu.pl/Badanie_PC/6/Rotation_arithmetic_S03.html.

Each category of geometric symbol (varying in terms of velocity scale and motion type) has been randomly positioned in twenty different locations of the administrative border map. As mentioned above, all dynamic map symbols were divided into five speed classes (according to selected motion distribution scales), and only one of dynamic symbols was the fastest one on the map. Figure 2 presents an example of map design (with pentagon symbol) with motion velocity distribution according to exponential scale. When it comes to the distribution of individual speed classes in terms of the velocity of geometry change, a random distribution was applied. ArcGIS PRO 3.1 software and the Create Random Points tool (based on Python) were utilized, enabling the random assignment of specific values to specific points distributed in geographic space.

To avoid displaying every type of geometric symbol on the same base map, a separate administrative boundary map has been designed for each symbol category (27 geometric variants). We placed geometric symbols randomly throughout the whole map, although we implemented certain guidelines to ensure an even distribution of symbols across the entire map. Firstly, we placed two symbols within each of the ten administrative units. However, if a particular administrative unit was too small to accommodate two symbols, we selected one of the larger units and placed three geometric symbols there instead. The described situation could be seen in Figure 2.

All administrative maps were developed based on real map units of level 2 (Nomenclature of Territorial Units for Statistics - NUTS 2). To ensure uniformity, areas of similar size were selected from different parts of the world, resulting in 10 neighboring administrative units on each map. We avoided units that were excessively elongated horizontally or vertically. All units were obtained in Shapefile format and converted into vector graphics for further processing.

### Apparatus

We used the SR Research EyeLink 1000 Plus eye tracker with sampling rate 2000 Hz (with additional chin and forehead rest) for recording participants’ eye movements. All materials were shown on a 21 inch monitor with 1920 x 1080 pixel resolution. The distance between participants’ eyes and the eye tracker was approximately 50 cm, and the distance between the monitor and participants’ eyes was around 90 cm.

### Procedure

The procedure consisted of presenting all 42 maps with dynamic point symbols in a random order to each participant individually. In the first group (G1), participants were shown only maps with symbols but no target symbol. In the second group (G2), participants were shown the target dynamic point symbol with actual speed parameters. The target symbol shown was always the fastest one. G1 was asked to find the fastest changing symbol, and G2 was asked to find the target symbol that preceded each map. G2 had unlimited time to study the target symbol displayed before each map. Before the actual experiment participants from both groups were calibrated with a 9-point calibration procedure. Then, they performed a set of familiarization tasks. The participants’ average gaze sample score was 98% ± 0.8%.

On each map dynamic symbols were animated for 1000 milliseconds. After this time all map symbols stopped and participant selected the target symbol from the map by clicking left mouse button. Before each stimulus presentation, participants were shown an interstimulus cue, which consisted of a 3-second time counter positioned at the center of the display. They were specifically instructed to fixate their gaze on this interstimulus cue.

## Results

**Figure 3.**
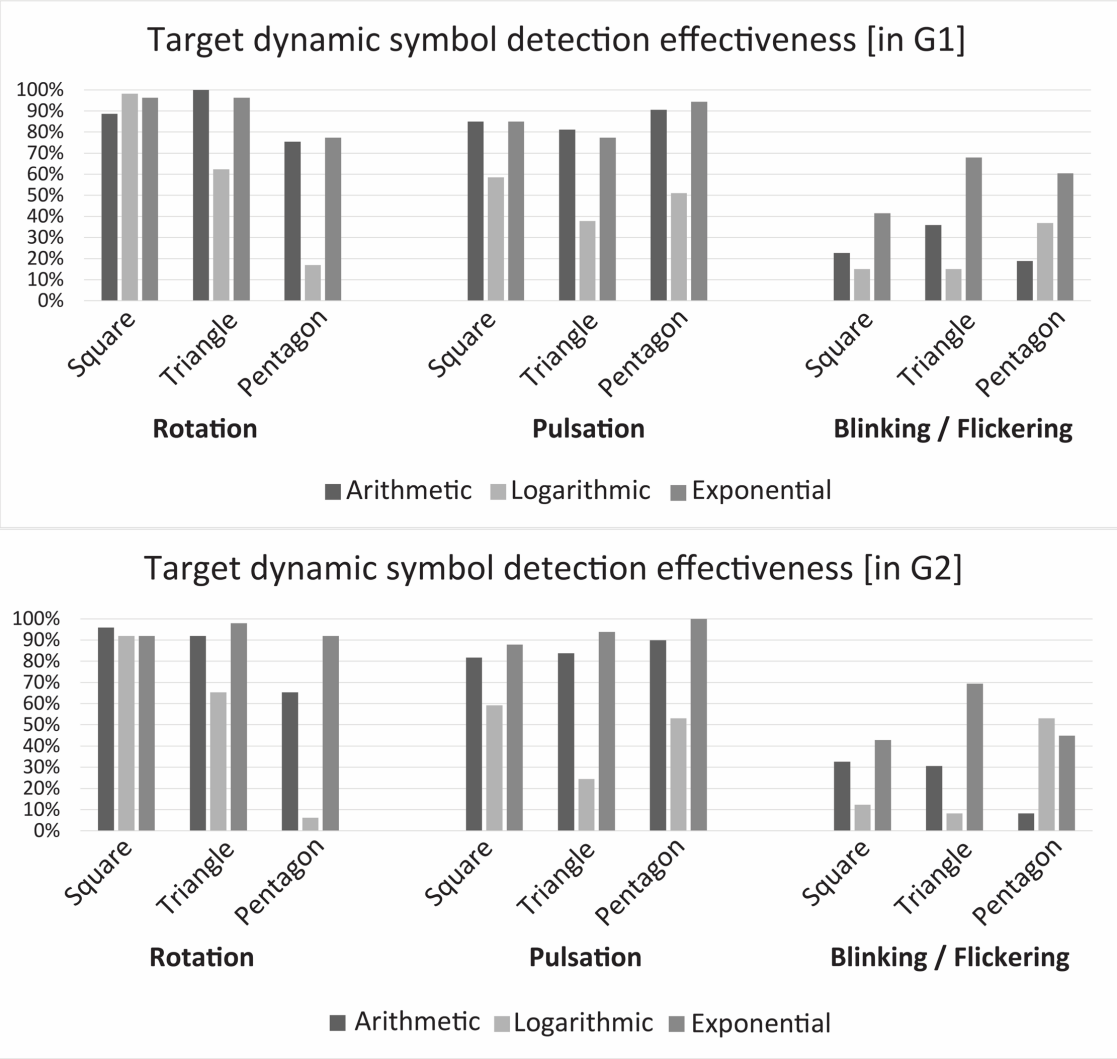
Detection effectiveness among geometric symbols with different movement type and motion distribution scales.

Effectiveness is the most basic metric that shows detection accuracy (10). This corresponds to the total percentage of correct detections of target symbol by all study participants. In both G1 and G2, detection of dynamic symbols in exponential scale was the most effective for geometric symbols. Arithmetic was the second most effective motion distribution scale. The logarithmic scale of motion distribution was the least effective. As far as motion type is concerned, the most effective detection was by rotation, and blinking/flickering was the least effective. However, pulsation was moderately effective except for the logarithmic scale for which detection effectiveness was rather low (between 38% and 58%). Therefore, the rotating square was the most effectively detected symbol within all three motion distribution scales. Figure 3 presents detailed results of detection effectiveness in both G1 and G2.

Secondly, symbol shape (3 conditions), movement type (3 conditions), and motion velocity distribution scale (3 conditions) were the within-subject factors. The evaluation of the visual process was based on several dependent variables, such as the number of total fixations, the number of fixations on target symbol, and time to the first fixation on the target. We used Box Cox transformation (2) to achieve normal data distribution. Therefore, the statistical analysis was based on Factorial ANOVA.

The measures of eye movement discussed above, which we analyzed, specifically pertain to the timing of the display initiation for the moving symbols (excluding the interstimulus period represented by a 3-second countdown). We did not perform eye analysis during the decision-making phase, that is, when the symbols were stationary. The decision to consider the total number of fixations was motivated by the fact that certain participants in the study provided correct answers without fixating on any symbol. In other words, they were able to identify the target symbol during preattentive vision, bypassing the need for deliberate visual fixation. From this perspective, and in our opinion, focusing solely on the time to the first fixation appears to be an incomplete analysis.

**Figure 4.**
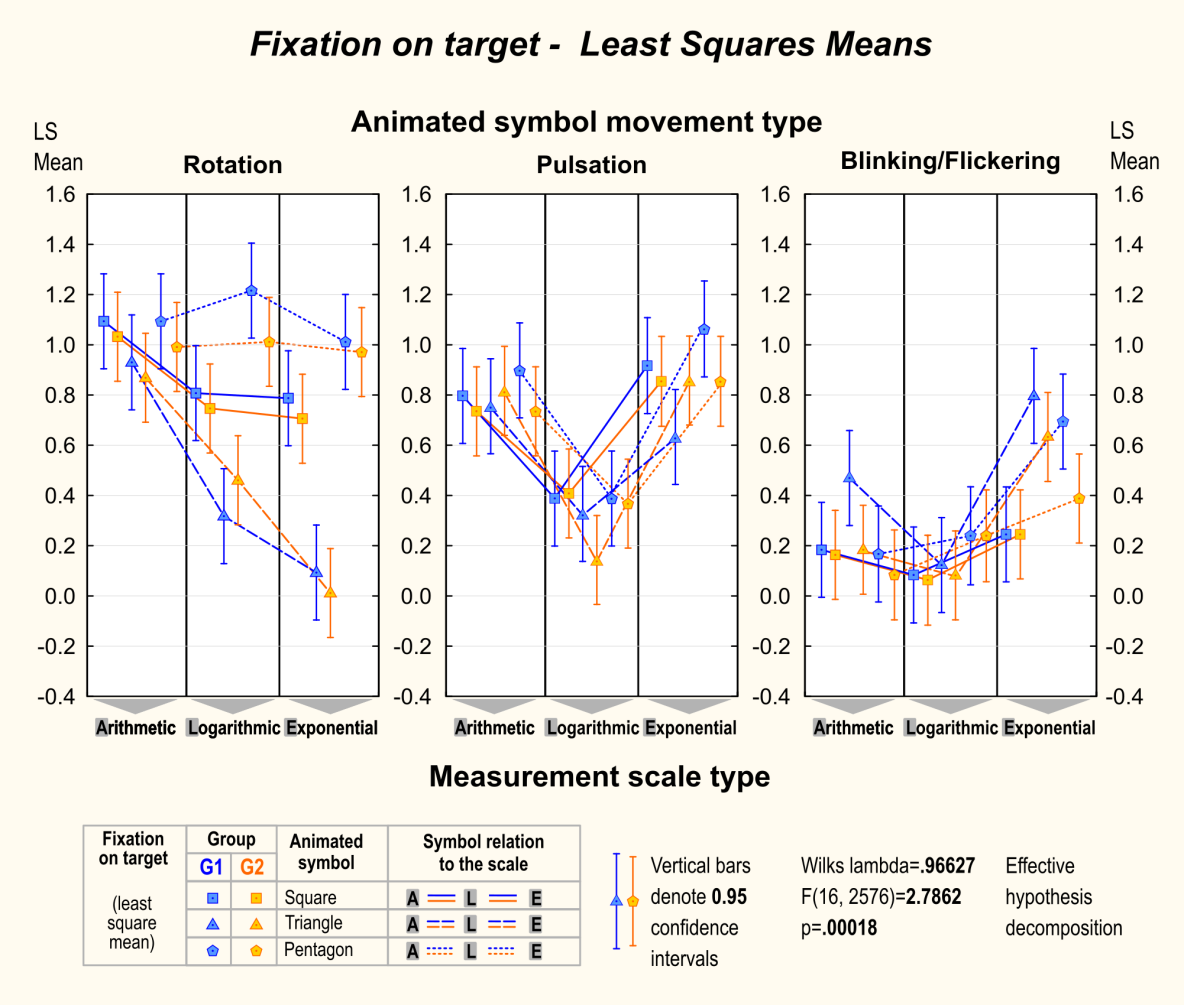
The average number of fixations on the target. Factorial ANOVA shows differences among different symbols with various motion types and scales.

**Figure 5.**
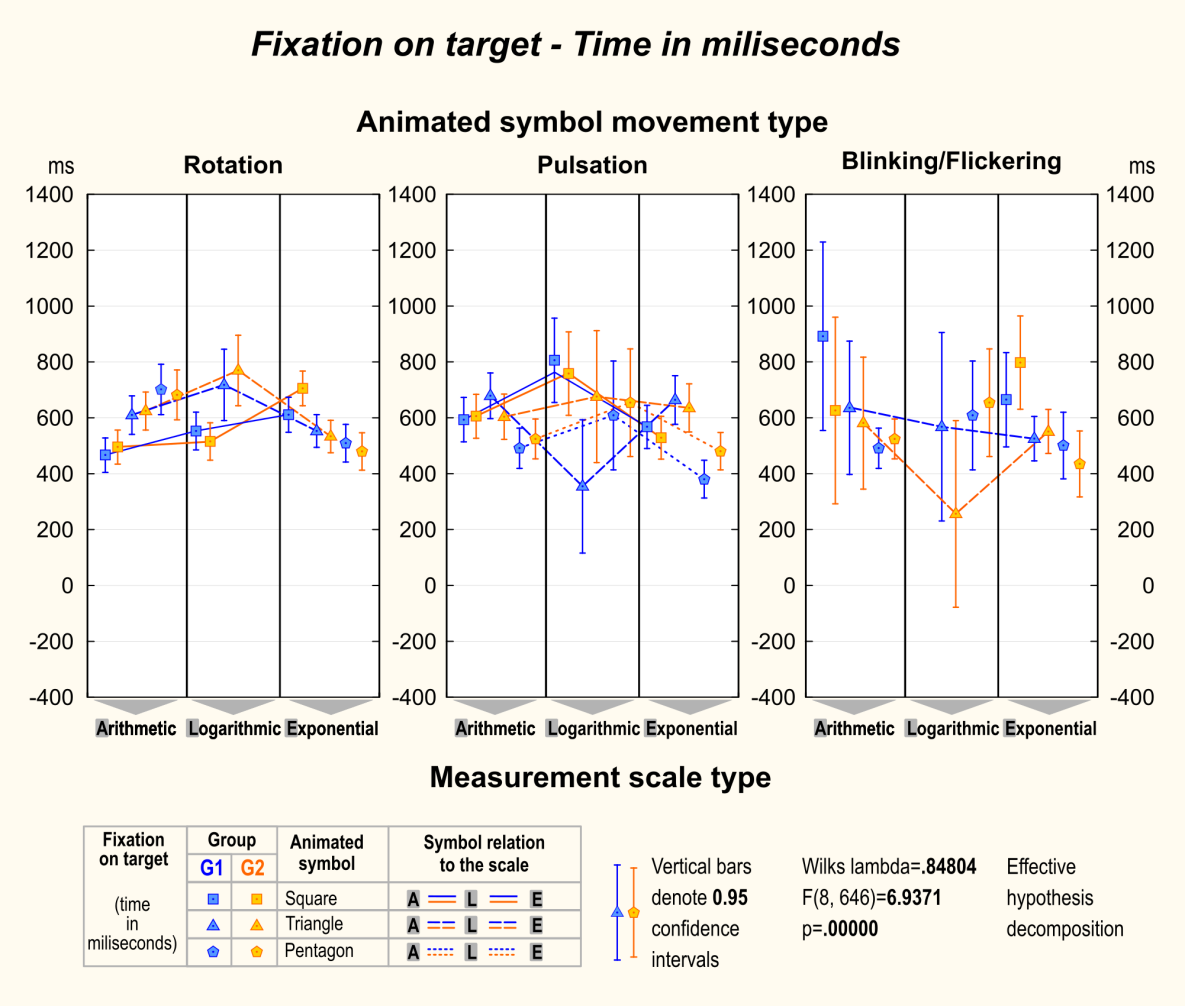
The average time to the first fixation on the target. Factorial ANOVA shows differences among different symbols with various motion types and scales.

The differences between G1 and G2 in the number of total fixations were not statistically significant among all factors. The only crucial significance was determined by movement type (F=18.531; p<.000001) and symbol shape separately (F=2.700; p<.029). It turned out that participants from G2 had slightly fewer fixations on the map then participants from G1 while using blinking/flickering and pulsating symbols (respectively in G2 3.0±1.3 fixation on average while blinking/flickering; 3.0±1.3 fixations on average while pulsating; in G1 3.5±1.4 fixations on average while blinking/flickering; 3.4±1.4 fixations on average while pulsating). Similar significance could be seen for symbol shapes. Participants from G2 had, in total, slightly fewer fixations than G1 (respectively for G2 3.1±1.3 fixation on average for squares; 3.3±1.3 fixations on average for triangles; 3.2±1.3 fixation on average for pentagons; respectively for G1 3.4±1.4 fixations on average for squares; 3.6±1.4 fixations on average for triangles; 3.6 fixations on average for pentagons). Figure 4 and 5 present the average number of fixations on the target and the average time to the first fixation on the target in research groups.

A factorial ANOVA revealed significant differences in the time to the first fixation among symbols, as depicted in Figure 5. Notably, in both groups during rotation, the square emerged as the symbol that received the fastest fixation, particularly in the context of arithmetic and logarithmic scales. However, of greater significance is the observation that in some instances, the initial fixations occurred beyond a 200 ms period following the commencement of symbol movement. This suggests that the target symbol was detected during the preattentive vision phase. This was particularly evident in the case of logarithmic scale and pulsating or blinking/flickering symbols.

**Figure 6.**
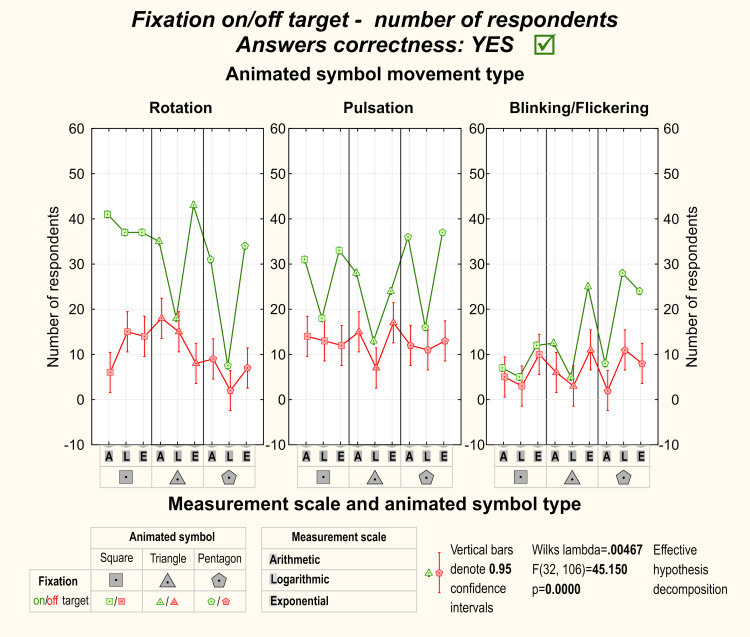
Total number of participants who detected and fixated the target symbol correctly.

Correct detection of the target dynamic symbol among distractors was related to fixation on the target. On the other hand, incorrect symbol detection was often related to fixation on the distractor rather than the target symbol. Even though participants that correctly detected the target dynamic symbol would fixate on it more frequently, some of them detected symbol correctly without fixating on the target. It was especially visible during rotation and pulsation. Figure 5 and 6 show detailed results of Factorial ANOVA with statistical significance among participants’ effective target detection.

**Figure 7.**
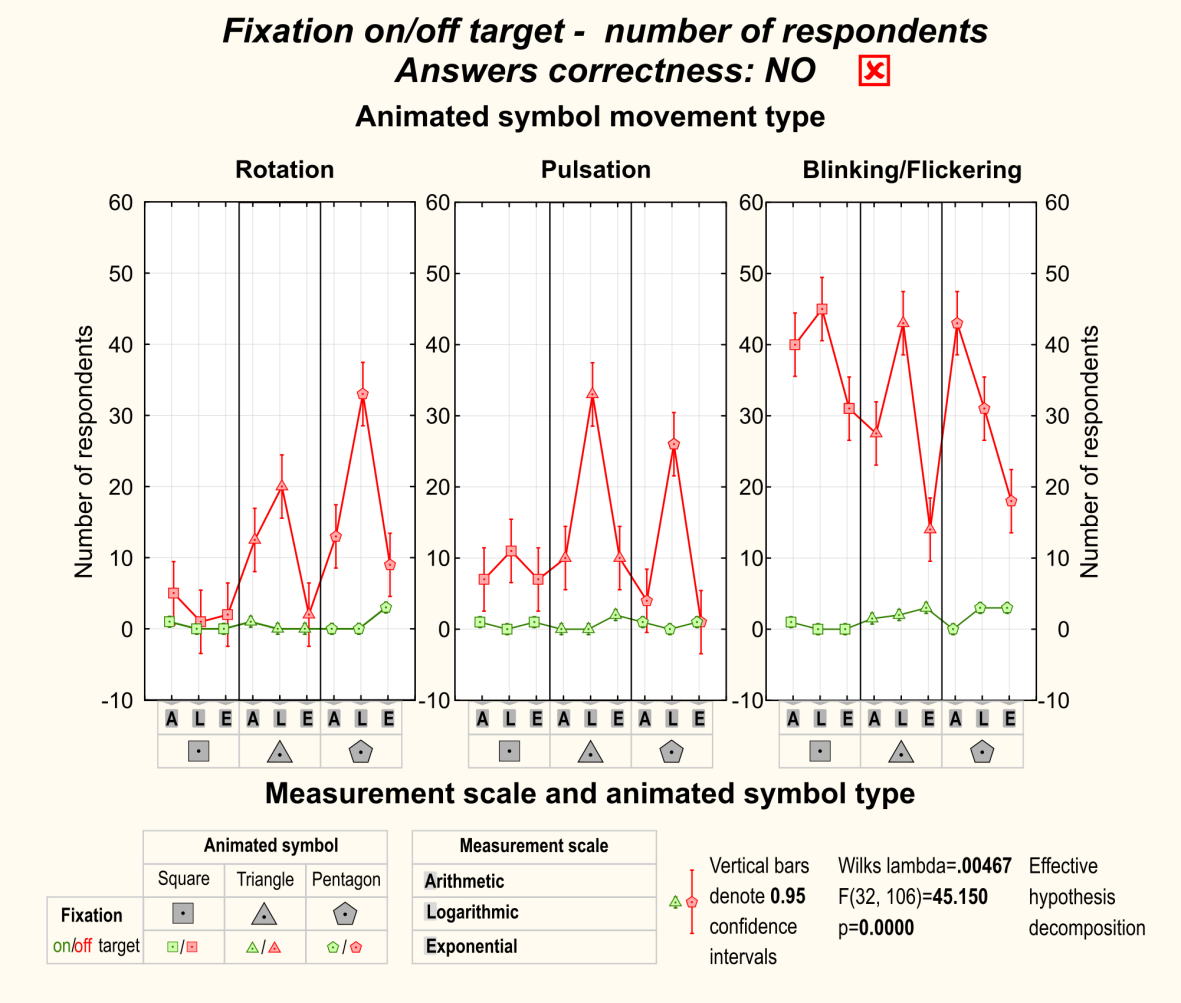
Total number of participants who detected and fixated the target symbol correctly and incorrectly.

Additionally, we conducted an analysis of the impact of point location (central or peripheral) on the accuracy of target symbol detection and the number of fixations. The ANOVA analysis did not reveal any significant differences in the number of fixations between centrally located points and those positioned peripherally. There were also no differences observed between the groups (G1 vs G2). The Pearson χ2 test did not provide significant evidence supporting the impact of target symbol location on the accuracy of detection between different types of symbols in both groups (p=.258 for G1 and p=.263 for G2). However, when considering different scales, we observed that peripherally located symbols were more frequently detected than centrally located symbols in both groups when using a logarithmic scale (50% correctly detected peripherally located symbols vs 30% correctly detected centrally located symbols) (p=.002). Nevertheless, the results for the logarithmic scale were lower compared to the other scales in both groups.

## Discussion

The study hypothesis was confirmed by the results. Motion velocity in dynamic geometric cartographic symbols is a feature that could be processed preattentively. However, the type of movement constitutes the most influential factor in this initial vision. Not all types of change in the symbols’ motion were processed preattentively. It includes blinking/flickering, which could be considered as continuous change of brightness. The motion velocity distribution scale would be the second most important factor. Especially, logarithmic distribution could be considered as non-supportive of preattentive search for the fastest symbols. It appeared that rotating square regardless of the motion distribution scale was the most effective for preattentive detection on the map for geometric symbols.

Distribution of motion velocity between symbol classes plays a significant part in proper cartographic design. However, Duncan & Humphreys (9) suggested that differences between the target and the distractor should be high enough for the user. We noticed that logarithmic motion distribution failed to support preattentive processing. It seems that there are small motion velocity differences between the target and distractors. In the map design process this kind of distribution should be avoided or at least the user needs to have more time to observe differences between symbol classes. However, in terms of symbol shape, square appears to be the most effective in preattentive vision and guiding attentive processing (38, 39). Blinking/flickering type of symbol change is not a preattentive feature among other distractors with the same motion. This observation complements the studies conducted by Tynan & Sekuler (36), Nakayama & Silverman (33), Driver et al. (8), and Huber & Healey (16), particularly in the context of mapping techniques employed in animated cartography. Velocity of motion in general is a preattentive feature. However, for dynamic map symbols, not all motion types are clearly preattentive despite the motion velocity distribution scale. Therefore, we suggest avoiding this type of motion in any scale since effectiveness results were relatively low.

Although previous studies (29; 6) have suggested that the specific location of a point plays a role in visual search, our research did not find any supporting evidence for this hypothesis. During the preattentive vision process, we did not observe any statistically significant effects based on the location of the target object. The only observed difference was in the logarithmic scale between centrally and peripherally located symbols. However, the results in the logarithmic scale were significantly lower compared to the other scales, indicating that this finding alone is not sufficient to draw a definitive conclusion. This finding aligns with the conclusions of Wolfe et al. (37), which stated that when the target differs from the distractors in a single feature, such as color or shape, it can be rapidly and effortlessly detected, regardless of its location. This phenomenon is commonly referred to as the "pop-out" effect (17).

The study presented is focused only on geometric symbols. For future studies, we would like to process the data obtained for pictorial symbols, and compare all the results. However, based on the results for geometric symbols, we would like to extend studies for more shapes. Compared to previous studies of this type, we did not involve a task with the absence of the target symbol. Our study focused primarily on one specific type of map usage, namely the search for the fastest changing symbol or the identification of the highest value. However, it is crucial to acknowledge that the potential of map usage scenarios extends beyond this particular task and includes various other types of tasks, such as symbol comparison. While comparison tasks between symbols are indeed common in map usage scenarios, the ability to quickly identify the highest value holds practical implications. In certain cases, the comparison task can be centered around the fastest symbol, which serves as a benchmark or reference point for evaluating other symbols. An example involves comparing how a given economy fares in terms of the strongest economies within a specific region. Future examination could also consider other methods of capturing map readers’ reaction (e.g. think-aloud protocols, mouse tracking, fMRI etc.) and possible combinations of them.

The presented research will contribute to more effective publishing of animated maps. First and foremost, this study reveals the significant role of the conjunction between symbols' shape and movement type in visual search for dynamic symbols on a map. Our findings highlight that the design of animated maps should not solely focus on the shape of symbols but also consider the specific type of movement associated with them. Hence, it is imperative to emphasize that the selection of appropriate movements on the map should not be arbitrary. Therefore, our recommendation for map designers is to utilize rotating squares as symbols. However, if different symbols such as rotating pentagons or triangles are employed, it is essential to apply arithmetic or exponential speed distribution scales. When considering the use of pulsating symbols, it is advisable to avoid implementing logarithmic speed distribution among different classes. Furthermore, it is recommended to refrain from incorporating blinking/flickering symbols. This last recommendation aligns with the studies conducted by Lai and Yeh (28). On this basis, methodology used for the development of dynamic point symbols can be established in detail. On the other hand, it contributes to the deeper understanding of the essence of preattentive processing of dynamic objects.

### Ethics and Conflict of Interest

The author(s) declare(s) that the contents of the article are in agreement with the ethics described in http://biblio.unibe.ch/portale/elibrary/BOP/jemr/ethics.html and that there is no conflict of interest regarding the publication of this paper.

### Acknowledgements

This research was funded in whole or in part by National Science Centre, Poland 2020/39/D/HS6/01993. For the purpose of Open Access, the author has applied a CC-BY public copyright license to any Author Accepted Manuscript (AAM) version arising from this submission.
